# Evaluation of Acute Toxicity and Antioxidant Response of Earthworm Exposed to a Lignin-Modified Crosslinked Hydrogel

**DOI:** 10.3390/toxics11060476

**Published:** 2023-05-24

**Authors:** Humberto D. Jiménez, Eulogio Orozco, Saira L. Hernández, Ana C. Ramírez, José M. Velázquez, Gilberto Velazquez, Amelia del C. Minjarez, Adalberto Zamudio, Milagros M. Flores, Sandra F. Velasco

**Affiliations:** 1 Chemistry Department, School of Exact Sciences and Engineering, University of Guadalajara, Blvd. Gral. Marcelino García Barragán 1421, Olímpica, Guadalajara 44430, Jalisco, Mexico; humberto.jimenez5529@academicos.udg.mx (H.D.J.); saira.hernandez@academicos.udg.mx (S.L.H.); ana.ranguiano@academicos.udg.mx (A.C.R.); jmiguel.velazquez@academicos.udg.mx (J.M.V.); gilberto.velazquez@academicos.udg.mx (G.V.); 2 Institute of Educational Management for Health and Society, Pablo Quiroga 289, Constitución, Zapopan 45180, Jalisco, Mexico; amelimin@igestioneducativa.org; 3 Department of Physics, School of Exact Sciences and Engineering, University of Guadalajara, Blvd. Gral. Marcelino García Barragán 1421, Olímpica, Guadalajara 44430, Jalisco, Mexico; adalberto.zojeda@academicos.udg.mx; 4 Medical Science, University of Colima, Avenida Universidad 333, Las Víboras, Colima 28040, Colima, Mexico; mflores55@ucol.mx

**Keywords:** modified hydrogel, acute toxicity, earthworm, antioxidant capacity

## Abstract

Hydrogels are polymers of great importance due to their multiple applications, which have led to an exponential increase in their production. However, once they have fulfilled their function, they become waste and their ecotoxicological effects are unknown. The aim of the present study was to evaluate the acute toxicity and total antioxidant capacity of the earthworm (*Eisenia fetida*) exposed to a terpolymeric hydrogel (acrylic acid, acrylamide, and 2-acrylamido-2-methyl-1-propane-sulfonic acid) crosslinked with modified kraft lignin. Four different amounts of hydrogel per unit area were evaluated (0.0924, 0.1848, 0.9242, and 1.848 mg hydrogel/cm^2^) plus a control, and three replicates were performed for each group. Starting from the amount of 0.1848 mg hydrogel/cm^2^, the earthworms showed physiological and behavioral alterations; at higher amounts, 0.9242 and 1.848 mg hydrogel/cm^2^, more acute signs were observed with mortality rates of 51.7% and 100%, respectively. On the other hand, the antioxidant activity assay showed that the higher the hydrogel exposure amount, the higher the oxidative stress, as evidenced by lower antioxidant activity (67.09% inhibition of the ABTS^●+^ radical). Therefore, we concluded that the lignin-modified hydrogel generated oxidative stress and acute lethal toxic effects in *Eisenia fetida*.

## 1. Introduction

Hydrogels are hydrophilic polymers in gel form that have a high capacity to retain water without dissolving, thus swelling and increasing their dimensions without deforming until reaching their maximum degree of hydration or swelling index [1,2]. When swollen, hydrogels are soft, elastic, have high affinity for water, and possess high thermal and mechanical stability as well as biocompatibility. These aspects that make them among the most versatile materials. When completely dry, they are known as xerogels [3]. These polymers have the enormous feasibility of adding different chemical groups to their structures, which provide them with specific characteristics that result in a wide range of gels. These characteristics make them attractive for applications in diverse areas, such as agriculture, biomedicine, biomedical engineering, pharmaceuticals, food, and more recently, environmental remediation [4,5].

Although many of these materials have been tested and have many applications, such as in the controlled release of molecules of interest [6], it is also true that they are not entirely compatible with the environment. They have low degradation rates since they are mostly made of acrylates, which are not biodegradable and cause accumulation problems in soil [7,8]. Conventional polymers can take tens to hundreds of years to degrade, depending on intrinsic and external factors. In the case of biodegradable polymers, the time ranges from 18 months to three years. Although this time is much shorter due to interaction effects with trophic elements, it is still significant. Therefore, it is extremely important to know the environmental impact of these materials as they degrade [9].

Nowadays the use of these polymers has increased, which has led to a progressive increase in discharges into the environment, causing negatively impacts and also unpredictable consequences. When these polymers are released into the environment, in addition to interacting with other chemicals, they can interact with trophic organisms. The environmental impact due to these materials is known to include the death of seabirds, mammals, fish, and reptiles as a result of ingestion [10]. It is worth mentioning that hydrogels are a type of polymer, which, depending on their composition, are considered plastics. Meanwhile, their residues can be called microplastics, which can be ingested by different invertebrate taxa, causing reductions in somatic and reproductive growth, deterioration of physiological processes, and deterioration of the functions of the antioxidant system [11,12].

To reduce the environmental impact of these polymers, several alternatives have been proposed, including the synthesis of hydrogels in which the commercial crosslinking agent is replaced by natural organic compounds, such as lignin. Lignin is a natural heterogeneous polymer of aromatic character and is a constituent of plants. It is an abundant source of renewable raw materials and its chemical multifunctionality offers a wide range of properties. All of these factors are advantageous from a structural point of view. The ease of obtaining and isolating plant biomass, its affordability at the industrial level, and its degradation time [13] make the applications and prospects of lignin quite promising. However, adding an organic compound to its structure does not imply that it is a nontoxic or inert material; hence the importance of performing ecotoxicological studies on these new materials.

Ecotoxicological evaluation involves, among other tests, the use of bioindicator organisms. Earthworms are the preferred choice for the terrestrial field due to their role in the continuous consumption of plant residues and organic matter, as well as the transformation of these materials by means of their digestion, thereby favoring the development and maintenance of the soil structure with their excrement. They help maintain soil porosity, improve fertilization, and are representative of the invertebrates that live in soil. In addition, they have the ability to accumulate soil pollutants in their tissues and are important food for many higher order organisms, thus providing a route by which pollutants can be transmitted through the different species of a biological community to higher levels. Therefore, the species *Eisenia fetida* is widely used in terrestrial ecotoxicology [14,15,16]. Ecotoxicological studies currently use standardized protocols for the quantification of biological responses, called biomarkers, that in some cases reflect effects at the molecular, physiological and behavioral levels of the biota and are very useful in environmental assessments [17,18]. Several molecular markers (detoxifying enzymes, chelating molecules, and antioxidant defenses) have been effectively evaluated in various species of annelids, either acutely or chronically [10,19,20].

The evaluation of the toxicity of chemical substances using earthworms includes two methods: (a) contact tests, which evaluate acute toxicity and surface effects, in which earthworms are exposed to filter papers impregnated with the element or chemical compound, either alone or mixed; and (b) soil exposure tests, which allow the study of chronic effects due to ingestion and exposure [21]. These tests show the local effects that occur in the area of the body that has been in contact with the product or the systemic and molecular effects. Events occurring in these systems are interpreted as biomarkers of health status, life expectancy, or disease risk, and they are valuable tools for assessing the exposure to pollutants (e.g., pesticides), providing early information about damage (lethal, growth, and reproductive effects) in earthworms [22]. Molecular biomarkers include the use of oxidative stress indices of an organism or population exposed to various pollutants [23,24]. It has been described that hydrogels can be ingested by different invertebrate taxa, causing reductions in somatic and reproductive growth, alterations in physiological processes, and impairment of antioxidant system functions [11]. The antioxidant system is important as it minimizes oxidative damage and restores redox homeostasis after alterations caused by exposure to pollutants [11]. Exposure to pollutants can increase the production of reactive oxygen species (ROS) and affect the normal redox state of the cell [25]. The generation of ROS is considered as one of the mechanisms involved in the cytotoxicity of these hydrophilic polymers in terrestrial species, manifested in damage at the cellular, developmental, and reproductive levels [26,27]. As part of their adaptive strategies, earthworms can induce physiological changes, such as increased detoxification activity, energy allocation, and changes in protein concentration, to cope with these ROS [28]. Cells have defenses that are produced during metabolism (enzymatic) and those that are obtained through the diet, especially in fruits and vegetables (non-enzymatic). The separate evaluation of each of the defense mechanism allows the deduction of inconsistencies that compromise cellular homeostasis. A comprehensive way to assess the state of an organism against these oxidizing species is through the total antioxidant capacity assay, which allows quantifying the capacity of a tissue, organ, or system to neutralize free radicals [29]. This assay is used to evaluate the resistance to ROS in the tissues of organisms exposed to pollutants under natural conditions or in laboratory tests [30].

Therefore, this research had the following objectives: (1) to characterize a terpolymeric hydrogel composed of acrylic acid (AA), acrylamide (AM), and 2-acrylamido-2-methyl-1-propanesulfonic acid (AMPS) crosslinked with modified lignin; (2) to evaluate the acute toxicity of the earthworm *E. fetida* exposed to the hydrogel; and (3) to determine the total antioxidant capacity of the earthworms exposed to the hydrogel.

## 2. Materials and Methods

### 2.1. Reagents for Hydrogel Synthesis

For the modification of alkaline kraft lignin (AKL) as a crosslinking agent, the following reagents were used: low sulfonate alkaline kraft lignin, acryloyl chloride, both from Sigma Aldrich, (Saint Louis, MO, USA), purity > 97%, tetrahydrofurane (THF, HPLC grade from Thermo Fisher Scientific Inc., Fair Lawn, NJ, USA,). For the synthesis of the hydrogel, 2-acrylamido-2-methyl-1-propanesulfonic acid (AMPS), acrylic acid (AA), (both purity > 99%, from Sigma-Aldrich, Saint Louis, MO, USA,), and acrylamide, purity > 99% (AM) from Sigma-Aldrich, Saint Louis, MO, USA, were used as monomers. Potassium persulfate and sodium bisulfite (both from Golden Bell, México, purity > 98%) were used as redox initiators.

### 2.2. Modification of Kraft Lignin

**Figure 1:**
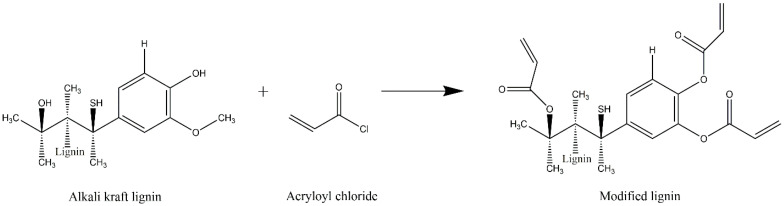
Scheme of reaction for the modification of alkali kraft lignin (AKL) with acryloyl chloride.

Alkali kraft lignin (AKL) modification was performed according to Rico et al. [31]. A solution was prepared with 2.5 g of AKL in 25 mL of distilled water with a stirring speed of 1000 rpm. Then, 5 mL of the solution was taken and transferred to a vessel, keeping the temperature in the range of 0–4 °C, stirring for 3 min at 900 rpm. Subsequently, acryloyl chloride was added dropwise and stirred for 30 min. Tetrahydrofuran (THF) was then added to the test tube and stirred vigorously until a homogeneous solution was obtained. The precipitate formed was transferred to a Petri dish and allowed to stand for 24 h. The precipitate was then collected and placed in an oven at 50 °C for 24 h. The modification reaction of the modified lignin with acryloyl chloride is shown in Figure 1.

### 2.3. Hydrogel Synthesis

The synthesis of the hydrogel was performed by free radical solution polymerization using potassium persulfate and sodium bisulfite as redox initiators and modified lignin as a crosslinking agent. Amounts of 0.78 g of AM, 4.72 g of AA, 4.52 g of AMPS (molar ratio of 0.5/3.0/1.0), and 0.5 g of modified lignin (5 wt.% relative to monomers) were dissolved in 16 mL of double-distilled water were placed in a glass reactor. Modified lignin and AM/AA/AMPS monomers were dissolved in double-distilled water. The solution was transferred to test tubes, and finally 0.01 g of each redox initiator, K_2_S_2_O_8_ and NaHSO_3_, was added to each tube with 900 rpm agitation, adding N_2(g)_ during polymerization. The tubes were then placed in a recirculator for 4 h at 55 °C, removed, and transferred to an oven at 50 °C for 7 days. The formed hydrogels, free of moisture, were washed several times with water until a pH of 5.6 was reached in order to remove possible traces of unpolymerized monomers. They were then placed again in an oven at 50 °C for 7 days for dehydration (xerogel formation). Once the xerogel was obtained, it was ground in a mortar and sieved to obtain a grain size of 600–1000 μm.

### 2.4. Hydrogel Characterization

#### 2.4.1. Elemental Analysis

The elemental analysis of the hydrogel was performed using a LECO Elemental Analyzer TruSpec Micro. In this test, 2 mg of sample was burned at 1273.15 K to obtain the weight percent composition of C, H, N, and S. The elemental analyzer was calibrated with ten tests using a sulfamethazine standard from LECO (51.8% C, 5.1% H, 11.5% O, 20.1% N, %11.5% S).

#### 2.4.2. Swelling Kinetics

One of the most important properties of a hydrogel is its absorption capacity, which is studied to observe the swelling behavior. The swelling kinetics were obtained from the swelling percentage (%H) of the synthesized hydrogel as a function of time, which was determined gravimetrically. The typical methodology used by some authors [31] consisted of immersing 0.1 g of xerogel with a grain size ranging from 600 to 1000 µm in 50 mL of distilled water (pH = 5.6) at 25 °C. The hydrogel was weighed at different time intervals; at each interval, excess water on the surface was removed with absorbent paper. This was carried out until no increase in swelling was observed, indicating that physicochemical equilibrium had been reached. The swelling percentage was calculated using Equation (1):

(1) $$\%H=\frac{W-W_{0}}{W_{0}}*100$$

where *W* is the mass of the hydrogel (g) at a time *t*, and *W*_0_ is the mass of the xerogel (g).

#### 2.4.3. Scanning Electron Microscopy (SEM)

The surface morphology was characterized using field emission scanning electron microscopy (FE–SEM) on a Tescan Bruker XFlash microscope (model MIRA LMU), using an accelerating voltage of 20 kV. The hydrogel samples were first dehydrated in a freeze dryer (10 N, Ningbo Scientz Biotechnology Co., Ltd. Ningbo, Zhejiang, China) with a residual pressure of 5 kPa at −50 °C. They were immediately coated with a gold layer for 30 s at 1 mA.

#### 2.4.4. Fourier Transform Infrared Spectroscopy (FTIR)

All FTIR spectra were collected with a resolution of 4 cm^−1^ in the range of 600–4000 cm^−1^ with a total of 32 scans using a NICOLET iS50 FT–IR spectrometer (Thermo Scientific) equipped with an ATR sampling unit (25 °C).

### 2.5. Organism of Study: Eisenia fetida

For the toxicity test, adult earthworms of the species *Eisenia fetida* with clitella (obtained from Lombricompost, Guadalajara, Jalisco, México) were used, according to the recommendations by the Organization for Economic Cooperation and Development (OECD). All adult earthworms had a well-defined clitellum and weighed from 250 to 400 mg. They were kept under dark conditions and at a controlled temperature of 21 °C ± 1 °C in culture chambers, maintaining extreme care in handling during all experiments.

### 2.6. Acute Toxicity Test

#### 2.6.1. Contact Toxicity Test (Contact Test)

The modified filter paper test was used [32]. First, the filter paper was moistened and hydrogel was deposited at different concentrations (0.0184, 0.0924, 0.1848, 0.9242, and 1.848 mg hydrogel/cm^2^) in order to determine the lethal amount for 100% of the earthworms. Then, the filter paper was left to dry at room temperature to avoid damaging the filter paper. Afterwards, the filter paper with hydrogel and the dry control were placed in a 9 cm Petri dish and 1 mL of water was added. After 30 s, one earthworm was placed per Petri dish. Before placing the earthworms in the Petri dishes, they were starved for 3 h. The samples were incubated in the dark at 21 °C ± 1 °C for 48 h. The morphological changes observed were recorded, as well as mortality after 48 h (considering as dead the organism that did not respond to any mechanical stimulus). Each experiment was performed in triplicate, respectively.

#### 2.6.2. Test on Artificial Substrate (PSA)

The artificial substrate was prepared by mixing the following ingredients: 10% peat, 20% clay, 70% industrial sand, and 1 g of CaCO_3_ to adjust the pH to 6.0 ± 0.5. The concentrations evaluated were 0 (control) 0.9242, and 1.848 mg hydrogel/cm^2^. The experimental units were containers with a capacity of 1 L, to which was added with 500 g of artificial substrate and the corresponding concentration of hydrogel, The substrate was mixed well and adjusted to 35% humidity. A total of ten adult worms with developed clitella, previously washed with distilled water, dried with absorbent paper, and weighed, were placed in the containers, which were covered with a cotton cloth to allow air flow. Humidity was adjusted to 35% every 7 days for 21 days. All of the containers were subjected to 12/12 h light/dark periods at 25–28 °C. Humidity and substrate pH were always sustained. Each trial had four replicates. Percentage of mortality after 21 days was evaluated, as reported by Shi et al. (2015) and Li et al. (2015).

### 2.7. Clinical Observations and Acute Toxicity Determinations

Observations were made on all Petri dishes at 24 and 48 h after contact with the substance. All signs of intoxication, abnormal behavior, growth inhibition, and mortality data were noted. The number of living organisms and sublethal clinical signs observed per individual were noted, including loss or reduction of movement, appreciable damage in the clitellum region, and presence of lumps in different areas of the body. These evaluations were carried out by emptying the Petri dishes on a surface and observing both the reaction of the earthworms to mechanical stimulus and their physical state. The test was considered valid if mortality in the control treatment did not exceed 10% at the end of the exposure period. The body weight of the worms was recorded at the beginning and end of the test to calculate the growth inhibition parameter, as reported by Jovana et al. [33], Li et al. [34], and Shi et al. [35], considering the following equation:

(2) $$IC=\frac{P_{0}-P_{t}}{P_{0}}*100$$

where *IC* is the growth inhibition, *P*_0_ is the initial weight (g), and *P_t_* is the total final weight (g).

In the case of the substrate test, the moisture content and pH of the artificial soil were measured, and the worms were weighed individually at the beginning of the test. Twenty-one days after the application of the hydrogel, the animals were counted and observed. Through the counting process, possible behavioral alterations as well as morphological changes were observed and considered. At the end of the study, the total number and weight of live worms per container were determined.

### 2.8. Euthanasia

At the end of the test, the surviving animals were sacrificed in an atmosphere of 20 dm^3^ min^−1^ of CO_2_ for 15 min.

### 2.9. Total Antioxidant Capacity Assay: ABTS^●+^ Radical Scavenging Activity of Earthworms Exposed to Hydrogel

The ABTS^●+^ (2,2′-azino-bis-(3-ethylbenzothiazolin-6-sulfonic acid) assay was performed according to Re et al. [36] with some modifications. The radical ABTS^●+^ was chemically generated using ammonium persulfate diluted in deionized water to a final concentration of 7 mM. The solution was diluted until reaching an absorbance of approximately 0.7 at 750 nm. In a 96-well microplate, 20 µL of the sample was mixed with 280 µL of the ABTS+ solution. The absorbance change (750 nm) was measured after 15 min of incubation at room temperature. The percentage of ABTS^●+^ radical scavenging activity was calculated according to Equation (3):

(3) $$AC\left(\%\right)=\frac{{Abs}_{c}-{Abs}_{m}}{{Abs}_{c}}*100$$

where *AC* (%) is the percentage of ABTS^●+^ radical scavenging activity, ${Abs}_{m}$ is the absorbance of the ABTS^●+^ solution with the sample, and ${Abs}_{c}$ is the absorbance of the ABTS^●+^ solution without sample.

### 2.10. Statistical Analysis

For both groups (control and treated), the percentage of mortality and the mean and standard deviation of the initial and final body mass were calculated. Furthermore, the masses of the animals in each treatment group were compared. To evaluate significant differences between treatments for the variables measured, after verifying the homogeneity of variance (Levene’s test), one-way analysis of variance (ANOVA) was performed with a significance level of 0.05, followed by least significant difference and Tukey’s post hoc tests to determine significant differences with respect to the control. All analyses were performed using IBM SPSS Statistics 27.0. Values of *p* < 0.05 were considered significant.

## 3. Results

### 3.1. Characterization of the Hydrogel Crosslinked with Modified Kraft Lignin

#### 3.1.1. Elemental Analysis Results

The exact composition of the terpolymeric hydrogel in wt.% (average for five tests) was 44.69% C, 5.03% H, 4.55% N, and 6.26% S (Table 1).

#### 3.1.2. Determination of Hydrogel Swelling

**Figure 2:**
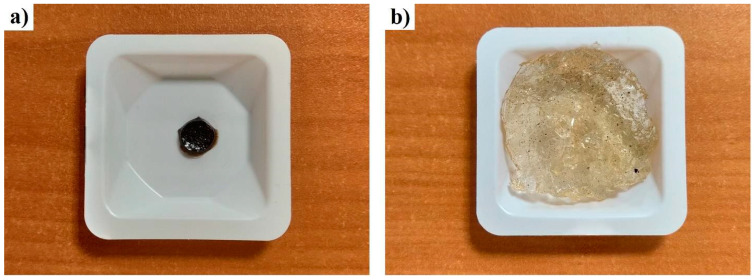
Swelling measurements: (**a**) xerogel, (**b**) hydrogel. The hydrogel prior to hydration (xerogel) and after hydration can be appreciated by observing its increase in size.

**Figure 3:**
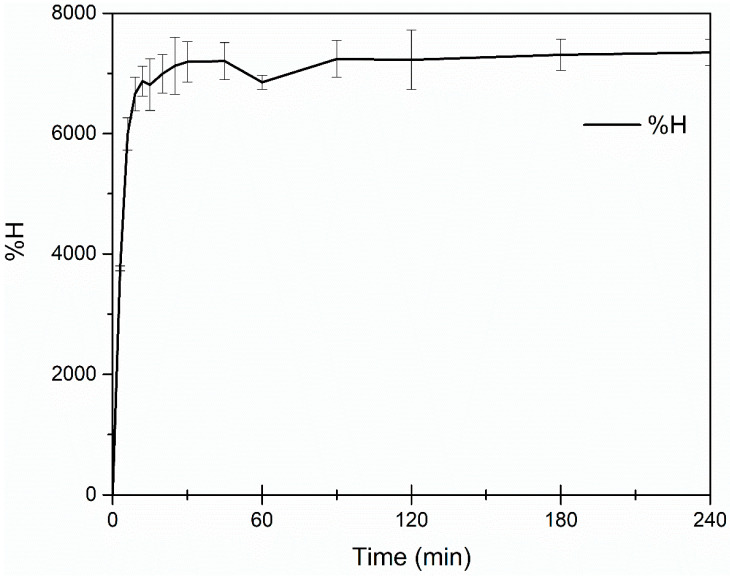
Swelling kinetics of terpolymeric hydrogel composed of acrylic acid (AA), acrylamide (AM), and 2-acrylamido-2-methyl-1-propanesulfonic acid (AMPS), crosslinked with modified kraft lignin, in distilled water at pH = 5.6.

The swelling property of a hydrogel (Figure 2) is due to the separation of the chains, given the chemical nature of the monomers, when in contact with some related substance; this is also possible thanks to crosslinking between the polymer chains, which prevents the hydrogel matrix from unraveling [2]. Figure 3 shows the maximum swelling of the hydrogel, which reached equilibrium with a maximum swelling of 7940% after three hours. Swelling has been reported to cause an improvement in the interaction of functional groups. For example, Qi et al. found that amino groups in hydrogels had antimicrobial properties and Shen, 2020, demonstrated that some hydrogels could function as antibacterial polymers [37,38].

#### 3.1.3. SEM Analysis

**Figure 4:**
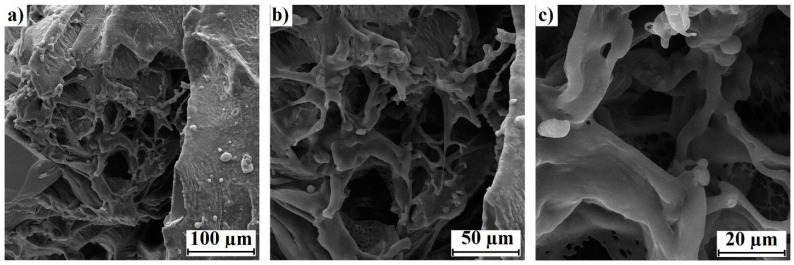
Three-dimensional network structure and porosity of the hydrogel. SEM images of the lyophilized hydrogel: (**a**) 100 µm, (**b**) 50 µm, (**c**) 20 µm.

The morphology of the lyophilized hydrogel was studied by SEM; the images obtained are shown in Figure 4a–c (from 100 to 20 µm, respectively). The three-dimensional network structure of the hydrogel was observed, demonstrating pores with irregular sizes ranging from approximately 50 to 20 µm. On the other hand, the three-dimensional network structure with obvious porosity is an important feature for absorption in hydrogels [39,40].

#### 3.1.4. FTIR Analysis

**Figure 5:**
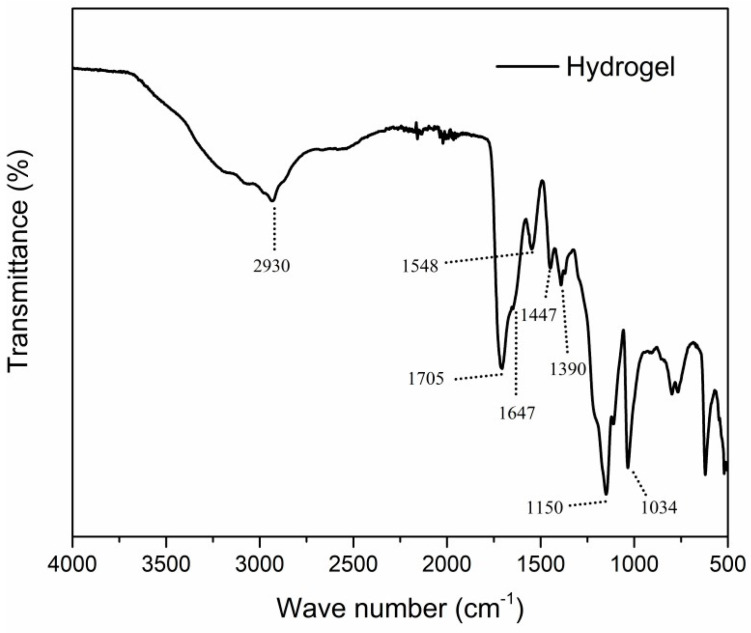
FTIR spectrum of the terpolymeric hydrogel crosslinked with modified kraft lignin.

In order to obtain a better understanding of the formation of the hydrogel structure, FTIR spectroscopy characterization was carried out. Figure 5 shows the spectrum obtained. The following characteristic absorption bands were observed: 2930 cm^−1^, assigned to O–H stretching vibrations [41,42]; 1705 cm^−1^, corresponding to C=O stretching of the carboxylic acid groups [25,41,43]; 1647 and 1548 cm^−1^, related to C=O and C–N stretching of the amide groups, respectively [25,41,42,44]; 1447 cm^−1^, related to scissor vibration of CH_2_ [41,45,46]; 1390 cm^−1^, corresponding to C–H stretching of the methyl group; 1150 cm^−1^, corresponding to S=O stretching of the sulfite group [41,47]; 1034 cm^−1^, related to C–O stretching of the carboxylic acid group [47]. The results shown for the hydrogel were typical absorption bands in a polymer of this nature [13,17,23,48,49,50,51].

### 3.2. Acute Toxicity Test on Earthworms (Contact Test)

#### 3.2.1. Test Validity

**Figure 6:**
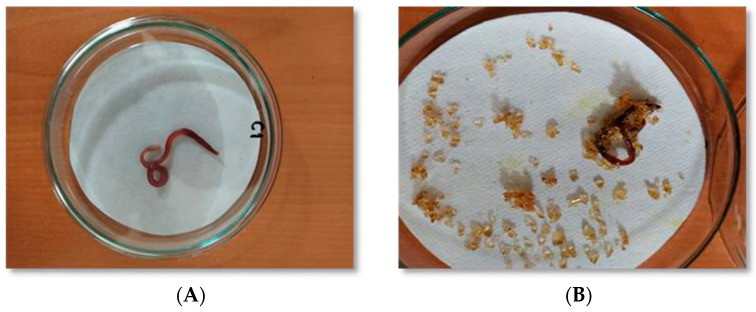
Physiological alterations. (**A**) Control group. (**B**) Group treated with hydrogel at the maximum amount tested. At higher amounts (0.9242 and 1.848 mg hydrogel/cm^2^), mortality and acute physiological effects were observed, such as loss of coelomic fluid, filiform appearance, and damage to the clitellar region.

The test was considered valid since there was no mortality in the control group (48 h) and the decrease in the average body mass of the earthworms in the control group was 14.57% (Figure 6A). These values were within the parameters established by international standards, which invalidated the test if the mortality in the control group was reported to be greater than 10% and if there was a 30% loss of average body mass [32,52].

#### 3.2.2. Mortality Rate

As a first result, it should be noted that the higher the test dose of the modified hydrogel, the higher the percentage of mortality and the greater the physiopathological changes observed in the exposed animals (Figure 6B). For the group exposed to the amount of 0.9242 mg hydrogel/cm^2^, the percentage of mortality was 51.7%, and for the group exposed to the amount of 1.848 mg hydrogel/cm^2^, mortality occurred in 100% of the organisms. Meanwhile, for the substrate test, the trial culminated with 100% survival.

#### 3.2.3. Growth Inhibition and Physiological and Behavioral Alterations

**Figure 7:**
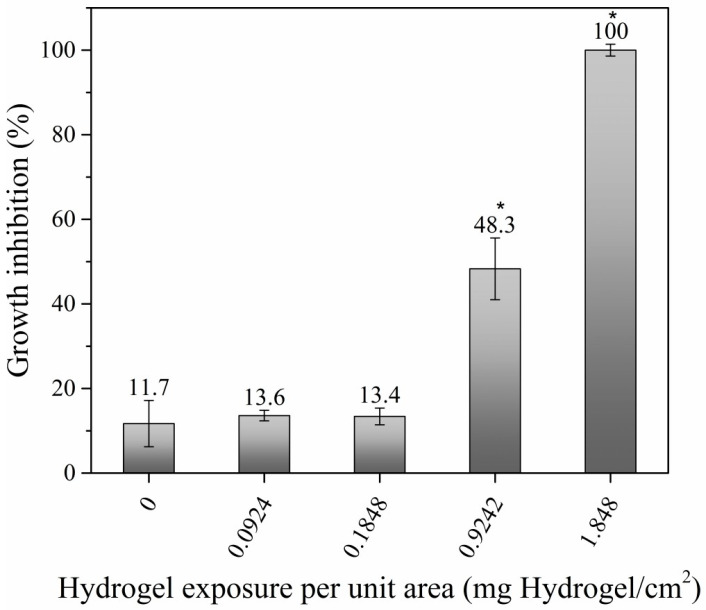
Percentage of growth inhibition at different hydrogel amounts per unit area: 0.0924, 0.1848, 0.9242, and 1.848 mg hydrogel/cm^2^, respectively. The * indicates the groups where there is a significant difference with respect to the control obtained by ANOVA (F (5,10) = 355.99, *p* < 0.001).

The groups exposed to the highest amounts of modified hydrogel (0.9242 and 1.848 mg hydrogel/cm^2^) presented 48.3% and 100% growth inhibition, respectively, which was statistically significant (Figure 7). These groups also presented manifestations of acute toxicity caused by contact with *Eisenia fetida* through the skin. The alterations presented increased not only in regularity but also in intensity, the most frequent being fluid loss, filiform appearance, damage to the clitellar region, and behavioral alterations (Figure 6B). The amounts of 0.0184, 0.0924, and 0.1848 mg hydrogel/cm^2^ did not cause growth inhibition or mortality; however, when the exposed organisms were compared with the controls, physiological and behavioral alterations were observed, indicating a positive amount–effect relationship.

When the test was carried out in artificial substrate, clinical alterations due to toxicity were detected in the group treated with the maximum concentration of hydrogel (1.848 mg hydrogel/cm^2^), including difficulty in digging since these worms remained in the lowest part of the containers in contrast to the worms of the control group. At the same time, coiling and formation of tangles were observed among several individuals because they had lower mobility compared to the worms of the control group. On the other hand, morphological changes were also observed, such as reduction of the clitellum, to the extent that it was no longer visible in some earthworms. Inflammation of the middle segments, segmental shrinkage, as well as a change in color were also observed, in contrast to the control group. The evolution of the body weight of the worms during the test is shown in Figure 7.

The control group showed the greatest loss of biomass, with a decrease of 55.5%. In contrast, the group treated with the maximum hydrogel concentration experienced a biomass decrease of 46.9%. Statistical comparison of the biomass variation between days 0 and 21 showed significant differences between the control and treated groups (*p* < 0.05).

#### 3.2.4. Total Antioxidant Capacity Assay: ABTS^●+^ Radical Scavenging Activity of Earthworms Exposed to Hydrogel

**Figure 8:**
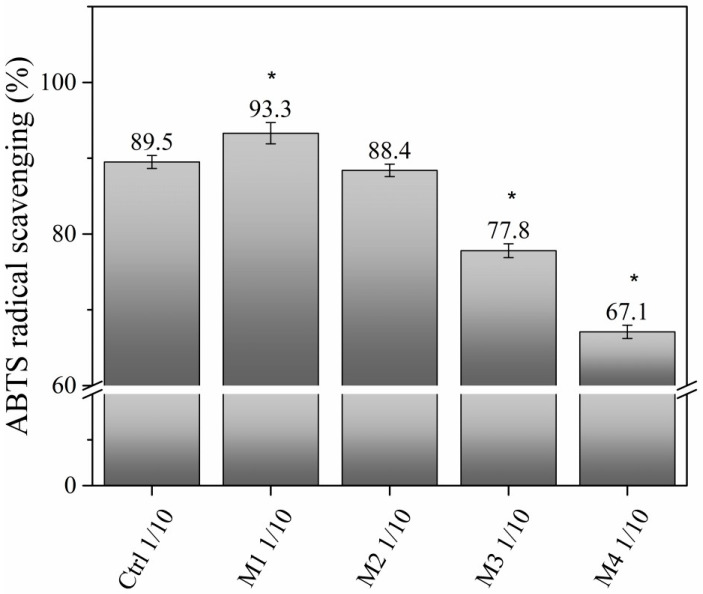
Percentage of inhibition of ABTS^●+^ radical. Percentage of ABTS^●+^ radical scavenging of 1/10 diluted control (Ctrl 1/10) vs. exposures to different 1/10 diluted amounts of hydrogel: M1, M2, M3, and M4 (0.0924, 0.1848, 0.9242, and 1.848 mg hydrogel/cm^2^, respectively). The * indicates the groups where there is a significant difference with respect to the control obtained by ANOVA (F (5,12) = 377.317, *p* < 0.001).

The values obtained for the ABTS^●+^ radical scavenging capacity are presented in Figure 8. When comparing the percentage of inhibition of the ABTS^●+^ radical from the control specimens with respect to the earthworms treated with different amounts of hydrogel, it was observed that the amount of 0.0924 mg hydrogel/cm^2^ induced the highest percentage of inhibition of the ABTS^●+^ radical, which was 93.33%. In contrast, the concentration of 1.848 mg hydrogel/cm^2^ was shown to induce the lowest percentage of ABTS^●+^ radical inhibition, at 67.09%. These values corresponded to the amounts of 48.45 and 60.35 µmol ascorbic acid/g sample, respectively.

When the percentages of ABTS^●+^ radical inhibition in substrate tests were compared, the control showed an activity of 98.73% while the 1.848 mg hydrogel/cm^2^ concentration induced 99.05% activity, which corresponded to the amounts of 33.35 and 61.93 µmol ascorbic acid/g sample, respectively) (Figure 8).

## 4. Discussion

Considering the significant number of products that can cause pollution, and given the variability of their nature and purpose, the need to know the ecological effects of substances released into the environment has been recognized in many countries.

Substances that have been modified to be more environmentally friendly, such as hydrogels crosslinked with organic materials, should also be considered. These are assumed to have a low environmental impact; however, such assumptions should be avoided to ensure that each product, whatever its origin and composition, is investigated from an ecotoxicological point of view prior to its release. In case such substances are already present in significant quantities in water and soil, their impact should be assessed. Since there are no mechanisms in nature for their efficient degradation, these substances become pollutant residues in water and soil, remaining in the environment temporarily or permanently and thus exposing and compromising soil organisms such as earthworms.

Earthworms play a key role in the maintenance of terrestrial ecosystems because they participate in the recycling of organic matter, fertilization, and soil protection against erosion processes, and they also play a crucial role in terrestrial ecotoxicological risk assessment [53,54,55]. Because they are very sensitive to pollution compared to other invertebrates [56], they have proven to be excellent bioindicators, thereby allowing adequate estimations of the toxicity and potential risks of pollutants in soil [56,57,58,59,60].

In the present work, the acute toxicity of lignin-crosslinked acrylic acid hydrogels was evaluated in the earthworm (*Eisenia fetida*). The results are discussed in the context of their use and interaction with the food chain.

The monomers selected for the synthesis of a terpolymeric hydrogel were acrylic acid, acrylamide, and 2-acrylamido-2-methyl-1-propanesulfonic acid, which are common components of hydrogels for agricultural use, due to their high capacity to retain moisture and fertilizers in the soil [51,61], and for scavenging heavy metals in water and soil decontamination [13,50,51]. These were crosslinked with the natural organic polymer lignin, which has a recognized potential in heavy metal absorption due to the variety of functional groups in its structure [62].

The synthesized hydrogel exhibited superabsorbent properties, with maximum absorption capacity values exceeding 1000 g of water per gram of xerogel. On average, hydrogels absorb up to 150 times their own volume, with a retention capacity of 980 mL of water L^−1^ [48,63]. Therefore, we obtained a good swelling efficiency in aqueous solution at an average temperature of 25 °C and a pH of 5.6, which is characteristic of industrial wastewater and contaminated land. The degree of swelling obtained in this work was much lower than that obtained by our team in previous studies [64] with AA-AM copolymer hydrogels (composition of 75:25 weight percent using ethylene glycol dimethacrylate (EGDMA) at 1% as the crosslinker), which achieved swelling of up to 70,000%. This was expected since, depending on the type and concentration of the crosslinker used, a tighter network may be generated that impacts its swelling capacity. On the other hand, the three-dimensional network structure showed evident porosity with irregular pore sizes ranging from approximately 50 to 20 µm, which is an important feature for absorption in hydrogels [39,40]. According to the above, the synthesized hydrogels showed characteristics making them promising in different fields, such as those already mentioned; however, their ecotoxicological effects were not favorable.

Upon exposure of *Eisenia fetida* to the hydrogel in the contact test, the stress suffered by the organisms was manifested by inflammation, bleeding, and death at the highest amounts tested (0.9242 and 1.848 mg hydrogel/cm^2^), with mortality rates of 48.3% and 100%, respectively. Effects on exposed earthworms [65] can be assessed with respect to growth inhibition, reproduction [61], or egg formation [50,66]. In the case of growth inhibition (%), significant physical effects were caused by contact through the skin of the earthworms exposed to the amounts of 0.9242 and 1.848 mg hydrogel/cm^2^; moreover, as exposure time elapsed, the earthworms presented elongation of the posterior part of the clitellum, a sign accompanied by segmented cuts and excessive movement at the slightest contact. It should be noted that hydrogels have superabsorbent properties and, when in contact with substances or an aqueous medium, are capable of retaining or releasing substances under specific conditions. In the case of this study, the hydrogel was in contact with the earthworm, the composition of which is 80% water, so dehydration occurred. However, the effects observed did not correspond only with this process. Effects similar to those observed in our study were found when *Eisenia fetida* was subjected to acute pesticide poisoning [67]. Similar damage was found in *Eisenia fetida* exposed by contact to sewage sludge with a high load of organic compounds for 48 h [68]. Analogous effects were reported by Roberts and Wyman Dorough in 1984 [69], who demonstrated, as in this study, that as the time of exposure to the hydrogel progressed, the effects significantly increased, beginning with coiling and the release of coelomic fluid, followed by stiffness and contracture, then continuing with the shrinkage of segments along the body of the earthworm, and ending with softening, poor muscle tone, and death within a few hours.

According to Lin et al. [70], the alterations suffered by earthworms upon contact with any pollutant or harmful organic compound are due to stress, especially of the oxidative type, whereby earthworms release enzymes for their protection. This was confirmed in this study, since we observed that the higher the amount of exposure to the hydrogel, the lower the antioxidant activity (67.09% inhibition of the ABTS^●+^ radical) of the earthworms. It should be noted that antioxidants are compounds that can inhibit or delay the oxidation of other molecules by quenching the initiation and/or propagation of free radical chain reactions [49].

For further studies, we propose to evaluate the biochemical responses of *Eisenia fetida* to the hydrogel through soil exposure tests, which in addition to allowing us to study the chronic effects derived from exposure and ingestion will provide information on the dynamics of the microbial community [71], changes in the ability of earthworms to form galleries, and agitation and removal of the pollutant in the soil [72]. For the characterization of the oxidative stress mechanisms generated in the earthworms due to exposure to the hydrogel, the quantification of a battery of enzymes involved in oxidative stress is contemplated. The evaluation of the behavior of hydrogels in the soil is also contemplated, since this usually depends on the external conditions to which they are exposed, such as changes in pH, solvent composition, ionic strength, light, temperature, and antigens, among others [73,74].

## 5. Conclusions

To date there are no reports of the determination of acute toxicity with *Eisenia fetida* in contact tests or the determination of antioxidant activity; therefore, the results obtained are of relevance in the field of biomarkers for further studies on environmental monitoring of the different types of hydrogels. Furthermore, it is important to highlight the need for the use of combined biomarker tests in the laboratory and field in order to extrapolate the information from the laboratory to the real ecosystem and thus predict the polluting and toxic potential of hydrogels.

## Figures and Tables

**Table 1:** Percent composition of C, H, N, and S in the hydrogel.

Sample	%C	%H	%N	%S
Synthesized Hydrogel	44.69	5.03	4.55	6.26

## Data Availability

Not applicable.
